# Influence of painless one-eye blindness on depression, anxiety and quality of life in glaucoma patients with a normal fellow eye

**DOI:** 10.1186/s12886-021-01845-2

**Published:** 2021-02-17

**Authors:** Gábor Holló, Nikolett Gabriella Sándor, Péter Kóthy, Anna Géczy

**Affiliations:** 1 Department of Ophthalmology, Semmelweis University, Mária u. 39, Budapest, 1085 Hungary; 2 Department of Personality and Clinical Psychology, Pázmány Péter Catholic University, Institute of Psychology, Budapest, Hungary; 3 Doctoral School of Psychology, University of Pécs, Pécs, Hungary

**Keywords:** Anxiety, Depression, Glaucoma-related blindness, Hopelessness, Quality of life, Psychometric testing

## Abstract

**Background:**

For clinical practice it is important to evaluate and compare anxiety, depression and quality of life of glaucoma patients with painless one-eye blindness and a normal fellow eye to unaffected age-matched individuals from a similar environment.

**Methods:**

Twenty-eight stable glaucoma patients (age, mean ± SD: 69.0 ± 13.3 years) with one normal and one painless blind eye, and 26 controls (age: 67.0 ± 14.0 years) completed the standard Hungarian adaptations of the Beck Depression Inventory, Beck Anxiety Inventory, Spielberger-Trait Anxiety Inventory, Hopelessness Scale, and Quality of Life Questionnaire SF-36 with the assistance of trained psychologist interviewers within 3 months after a detailed ophthalmological examination.

**Results:**

The groups did not differ in age, gender distribution, number of children, grandchildren and people in their household (*p* ≥ 0.235). The best corrected visual acuity (BCVA) of the diseased eye was minimal (median: 0.00), while BCVA of their better eye (median: 1.0) did not differ from that of the control group (*p* ≥ 0.694). Compared to the control group, the patients’ scores were significantly higher for depression (*p* ≤ 0.01), cognitive and psychophysiological symptoms of anxiety (*p* ≤ 0.05) and hopelessness (*p* ≤ 0.013), and lower (worse) for physical function, vitality, general health and bodily pain (*p* ≤ 0.045). No difference was found between the groups for mental health, physical role functioning, emotional role functioning and social role functioning (*p* ≥ 0.117).

**Conclusion:**

Our results show that patients with glaucoma-related one-eye blindness may require regular psychological support even when the visual performance of the fellow eye is fully maintained on the long run, and the patients’ everyday functioning is normal.

## Background

Glaucoma is a progressive optic neuropathy, one of the commonest causes of irreversible blindness, worldwide [1, 2]. Based on the different pathomechanisms several clinical forms of glaucoma can be distinguished, but in almost all types the development of severe and irreversible deterioration of the visual functions (visual field and visual acuity) takes several years; effective treatment requires regular control examinations; thus effective patient - ophthalmologist cooperation and good treatment adherence (instillation of the prescribed intraocular pressure lowering eye drops and participating in the scheduled visits) are mandatory [3–5].

It has been established that quality of life (QL) of glaucoma patients may significantly worsen due to fear of future blindness [6–8]. Significantly reduced QL due to anxiety was found in 30 to 64% of under-treatment glaucoma patients [9–11], and depression is present in 10.9 to 30% of glaucoma patients [10, 12, 13]. These psychological alterations are independent from the glaucoma patients’ ethnicity [11, 13].

Psychological alterations associated with glaucoma can be caused by the actual deterioration of the visual functions, but may also be induced by the possibility of future functional decline (glaucomatous progression) [14]. In the current study we investigated if increased anxiety, depression and decreased QL are present in glaucoma-related one-eye blindness, in which chronic painless blindness is present in one eye, but the visual functions of the fellow eye are normal, and a preventive laser intervention and/or regular eye examinations make the possibility of glaucoma-related future functional decline of the fellow eye unrealistic.

## Methods

All participants were treated in accordance with the Hungarian Psychological Association Ethical Codes. This study was approved by and carried out in accordance with the recommendations of Psychology Research Guidelines of the Ethical Committee of the Pázmány Péter Catholic University (United Ethical Review Committee for Research in Psychology registration number 58/2015.). Written informed consent from each participant was obtained before enrollment, in accordance with the Declaration of Helsinki. All applicable institutional and governmental regulations concerning the ethical use of human volunteers were followed. The investigation was conducted between January 2016 and December 2017.

The eye examinations were performed by glaucoma specialists. Patient screening and questionnaire examinations were conducted by a psychologist supervised by a clinical psychologist. The questionnaires were taken by trained graduate psychology students. The glaucoma patients were recruited in the Glaucoma Unit of the Department of Ophthalmology of Semmelweis University. The control participants were recruited by online invitation. Participation in the study was anonymous, on a voluntary basis; the participants did not receive compensation for their participation, and their further treatment was not affected in any way by the study. All participants were white Europeans.

Our cross-sectional investigation involved age, sex and education matched glaucoma patients and non-glaucomatous control subjects older than 18 years of age (Table 1). To be eligible for the investigation the glaucoma patients had to have one painless glaucomatous eye with severely impaired visual functions (“blind eye”) for which the best corrected visual acuity (BCVA) was less than 0.1 and the visual field was undetectable for more than 1 year with the low vision test of the Octopus perimeter; a fellow eye with normal visual functions (reproducible and normal 30 degree Octopus G2 visual field test with false positive and false negative response rates less than 20%, respectively), BCVA ≥1.0, a normal optic nerve head, and intraocular pressure consistently less than 22 mmHg with or without intraocular pressure-lowering medication; and the patients had to be regularly controlled in the Glaucoma Unit of the Semmelweis University (regular 3-month or 6-month visits). The eligible causes of one-eye blindness comprised all types of glaucoma and any glaucoma related/associated painless ocular disease. The normal fellow eye had to be free from any clinically significant disease. In primary angle closure glaucoma a preventive laser iridotomy of the normal fellow eye at the time of the diagnosis was not considered as a criterion for exclusion. The glaucoma patients were allowed to regularly use intraocular pressure lowering eye drops and artificial tear drops for dry eye disease. The characteristics of the glaucomatous blind eyes and the fellow eyes are shown in Table 2. The non-glaucomatous control participants underwent a detailed ophthalmological examination in the Glaucoma Unit of the Semmelweis University. They had to have no severe eye complaints before the study and at the time of the investigation, BCVA ≥1.0, clear optical media and normal optic nerve head on both eyes. Previous uncomplicated cataract and corneal refractive surgery, a clinically non-significant early cataract, and use of artificial tears for mild dry eye disease were allowed for the control participants since these conditions are common in the elderly population, and do not negatively influence QL. The characteristics of the normal eyes of the control subjects are shown in Table 3. All participants had to be free from any prior or current abnormal mental condition. Of the 36 eligible glaucoma patients 28 patients completed the psychological tests. The number of matched control subjects who completed all tests was 26.

**Table 1:** Demographics of the participants

	Glaucoma group (***n*** = 28)	Control group (***n*** = 26)
Age (year; mean, SD)	69.0 (13.3)	67.0 (14.0)
Sex (male/female)	13/15	13/13
Frequency of regular eye check (3/6-month)	23/5	NA
Systemic diseases (n)		
No disease	8	15
Cardiovascular	15	11
Diabetes mellitus	3	1
Myasthenia gravis	1	0
COPD	1	0
Osteoporosis	2	0
Sturge-Weber syndrome	1	0
Hyperthyreosis	0	1
Chron’s disease	0	1

*NA* Not applicable

**Table 2:** Characteristics of the blind glaucomatous eyes and their fellow eyes

	Glaucoma group, blind eye (***n*** = 28)		Glaucoma group, fellow eye (***n*** = 28)	
BCVA (median, quartiles)	0.00		1.0	
	0.00 0.01		1.0 1.0	
Duration of blindness (years, median, quartiles)	4.50		NA	
	4.50	20.25		
Primary reason of glaucoma-related/associated blindness				
Glaucomatous optic disc damage	18		NA	
Corneal scarring	2		NA	
Myopic retina degeneration	2		NA	
Retinal vein occlusion	1		NA	
Chronic retinal detachment	5		NA	
No disease	0		15	
Ocular hypertension	0		12	
Mild and stable glaucoma	0		1	
Number of daily glaucoma eye drop instillations				
No instillation	11		15	
1 instillation	6		10	
2 instillations	5		0	
3 instillations	2		3	
4 instillations	2		0	
5 instillations	2		0	

*BCVA* Best corrected visual acuity, *NA* Not applicable

**Table 3:** Characteristics of the right and left eyes of the control subjects

	Control group, right eye (***n*** = 26)		Control group, left eye (***n*** = 26)	
BCVA (median, quartiles)	1.0		1.0	
	1.0	1.0	1.0	1.0
Eye diseases (n)				
Mild or moderate myopia	7		8	
Hypermetropia	3		5	
Previous corneal refractive surgery	2		2	
Mild cataract	1		1	

*BCVA* Best corrected visual acuity

### Psychometric tests

Within 3 months after the eye examination the participants completed a psychometric test battery. This comprised five standard questionnaires, all validated for the Hungarian population (Table 4). The first questionnaire addressed sociodemographic characteristics: male/female gender, age (year of birth), education level (0–3 grades on the Likert-scale), marital status (unmarried, married, divorced, widow/widower, single, living with a partner), the number of children and the number of grandchildren and great-grandchildren. To measure anxiety two questionnaires were used. Of them, the Hungarian version of the Beck Anxiety Inventory (BAI) [15, 16], was completed first. BAI comprises 21 items and measures the cognitive and psychophysiological symptoms of anxiety. The Hungarian version of the Spielberg Trait Anxiety Test (STAI) [17, 18] was completed afterwards. STAI comprises 20 items principally addressing the emotional characteristics of anxiety. Then the Beck Depression Questionnaire (BDI) was completed to quantitatively assess the presence or absence of depressive symptoms [19, 20]. In the current investigation the full 21-item version of BDI was used. To evaluate the feeling of hopelessness the Hungarian version of the Beck Hopelessness Scale (BHS, 20-item) [21, 22] was used. The last questionnaire was the 36-item Short Form Health Survey (SF-36), which is designed to measure QL [23, 24]. The SF-36 questionnaire addresses 8 different factors, separately. The p*hysical functioning subscale* measures the physical limitations experienced by an individual in everyday activity. The *physical role functioning subscale* is used to measure the performance in routine tasks as a function of physical health (reduction of time devoted to routine tasks). The e*motional role functioning* factor measures difficulty in performing the individual’s everyday activity as a function of mental health, the decrease in the amount of effort and time devoted to these activities. The *vitality* subscale measures levels of fatigue and exhaustion, but it also measures vitality and enthusiasm. The *mental health* dimension measures irritability, resentment, sadness, calmness, and happiness. The *social role functioning* scale measures change in the intensity of relationships with relatives and friends as a function of physical health. The *bodily pain* factor measures experience of physical pain and its limiting effect on everyday life. Finally, the *general health* subscale quantifies general health status and attitude towards health.

**Table 4:** Tested characteristics used in the current analysis

Tested characteristics	Test	Original publication	Hungarian adaptation
Psychophysiological and cognitive symptoms of anxiety	Beck Anxiety Inventory (BAI)	Beck et al. 1988 [15]	Perczel Forintos et al. 2001 [16]
Trait anxiety	Spielberger-Trait Anxiety Inventory (STAI)	Spielberger et al. 1970 [17]	Sipos et al. l978 [18]
Depression	Beck Depression Inventory (BDI)	Beck et al. 1961 [19].	Kopp et al. 1990 [20].
Hopelessness	Beck Hopelessness Scale (BHS)	Beck et al. 1974 [21]	Perczel Forintos et al. 2001 [22]
Quality of life	SF-36 Quality of Life Questionnaire	Ware et al. 1992 [23]	Czimbalmos et al. 1999. [24]

### Statistics

The SPSS 24.0 software package was used for statistical analysis. The data distribution was tested for normality using the Shapiro-Wilk test. For data not normally distributed, the median values and the quartiles are shown, and the Mann-Whitney U test was used to compare the groups. Normally distributed data are presented as mean ± SD, and were compared between the groups with the unpaired t-test. The Chi square test was used to compare distributions between the groups. *P*-values less than 0.05 are considered statistically significant.

## Results

### Sociodemographic characteristics

The ages of the 28 glaucoma patients (69.0 ± 13.3 years) and the 26 control subjects (67.0 ± 14.0 years) were similar (unpaired t-test, *p* = 0.439), and no difference was found in the gender distribution between the groups (Chi square test, *p* = 0.793). No difference was found between the groups in the number of children (*p* = 0.783), number of grandchildren (*p* = 0.235), education level (*p* = 0.710) and family status (*p* = 0.34). There was no difference in BCVA between the better eye of the glaucoma patients and the right and left eyes of the control subjects, respectively (Mann-Whitney U test, *p* = 0.304 and 0.279). In the glaucoma group the median duration of severe functional impairment of the worse eye was 4.5 years (quartiles: 4.5 and 20.25 years, Table 2).

### Anxiety, depression and hopelessness

The score for cognitive and psychophysiological symptoms of anxiety was almost significantly higher (worse) in the glaucoma group (BAI, Mann-Whitney U test, *p* = 0.050). However, no significant difference was found for the affective symptoms of anxiety (STAI, *p* = 0.761; Table 5). In the glaucoma group significantly higher (worse) scores were found for both depression (BDI, *p* = 0.004) and hopelessness (BHS, *p* = 0.013) than in the control group (Table 5).

**Table 5:** Comparison of the glaucoma group and the control group for the tested psychometric parameters

	Glaucoma group (median, quartiles, ***n*** = 28)		Control group (median, quartiles, ***n*** = 26)		***P***-value^**a**^
Beck Anxiety Inventory (BAI)	8.00		3.00		0.050
	4.00	18.25	0.00	15.00	
Trait Anxiety Inventory (STAI)	36.50		39.00		0.716
	32.25	44.75	32.00	49.25	
Beck Depression Inventory (BDI)	6.50		2.00		0.004
	3.00.	11.75	0.00	5.25	
Beck Hopelessness Scale (BHS)	10.00		8.00		0.013
	8.00	11.00	6.00	9.75	
SF-36 physical functioning	90.00		100.00		0.004
	70.00	95.00	90.00	100.00	
SF-36 physical role functioning	100.00		100.00		0.152
	81.25	100.00	100.00	100.00	
SF-36 emotional role functioning	100.00		100.00		0.594
	100.00	100.00	100.00	100.00	
SF-36 vitality	70.00		77.50		0.045
	60.00	75.00	65.00	90.00	
SF-36 mental health	78.00		84.00		0.117
	61.00	88.00	76.00	100.00	
SF-36 social role functioning	100.00		100.00		0.600
	87.50	100.00	84.38	100.00	
SF-36 bodily pain	70.00		90.00		0.005
	57.50	97.50	87.50	100.00	
SF-36 general health	50.00		70.00		0.001
	40.00	68.75	65.00	81.25	

*SF* Short Form Health Survey

^a^ Mann-Whitney U test

### Quality of life

Compared to the healthy controls significantly lower (worse) scores were found for physical functioning (Mann-Whitney U test, p = 0.004) and bodily pain (*p* = 0.005) in the glaucoma group (Table 5). The glaucoma patients rated their vitality (*p* = 0.045) and general health (*p* = 0.001) significantly worse than the control persons (Table 5). We did not find significant differences between the groups in physical role functioning (*p* = 0.152), emotional role functioning (*p* = 0.594), mental health (*p* = 0.117) and social role functioning (*p* = 0.600; Table 5).

## Discussion

In the current cross-sectional investigation glaucoma patients with a glaucoma-related blind or almost blind eye and a fellow eye with normal function and no risk for visual function deterioration were investigated with various quantitative psychometric questionnaires for anxiety, depressive symptoms, physical and emotional roles, bodily pain experience, social role, and mental and general health feeling. The results were compared to those obtained on an age-, sex- and education-level matched non-glaucomatous control group with no significant eye disease. The background of our investigation is that glaucoma patients are known to have an increased prevalence of anxiety and depression [10, 25], and their QL is reduced [14]. These negative psychometric deviations can be caused both by the experienced visual impairment [26] and by anxiety about a potential future worsening of the visual functions [7, 27]. These results are not unexpected since most forms of glaucoma cause chronic, progressive and irreversible visual impairment, may decrease visual acuity, and affect both eyes.

In the current investigation the glaucoma population differed considerably from those investigated in the previously published studies. In our glaucoma group all patients had one blind or almost blind eye, and a fellow eye with normal visual field and normal BCVA which did not differ from that determined for the right and left eyes in the control group, respectively. Furthermore, any future glaucoma-related functional decline in the better eye was unrealistic since either the risk factor was preventively eliminated (e.g. preventive laser iridotomy was performed for occludable anterior chamber angle), or the intraocular pressure was well controlled with no or minimal topical medication, and the eyes were regularly checked at 3 to 6-month intervals including visual field progression analysis, for several years. All blind eyes were painless and their status had remained unchanged for several years before the current investigation. Thus, significant visual impairment did not represent a recent mental burden for the patients. Ophthalmologists treating similar patients may not assume any clinically significant, glaucoma related decline of QL since it is not justified by the binocular visual functions. However, to prove or disprove this assumption evaluation of the relevant psychometric functions is necessary.

It is important to emphasize that all standard psychometric tests used by us were previously validated for the Hungarian population. The test completion was assisted by professional interviewers, and no participant suffered from any psychological/psychiatric disease. In the control group approximately half of the participants had a refractive error that required spectacle correction, two individuals had previously undergone corneal laser surgery to correct their refractive errors, and one person had mild cataract. These data show that the control subjects were not free from ophthalmic abnormalities that frequently occur in their age group, and several of them had previously undergone ophthalmic examinations. Thus, a potential eye examination-related negative emotional reaction could be present also in the control group.

Comparing psychometric parameters between the participant groups, we found that depressive symptoms, psychophysiological and cognitive symptoms of anxiety, and experiencing hopelessness in everyday life all showed significantly higher (worse) scores in the glaucoma group than in the control group. Furthermore, the scores for physical functioning, vitality, bodily pain during work, and feeling of general health were also significantly worse for the glaucoma patients than in the control group. In contrast, physical role functioning, emotional role functioning, mental health and social role functioning did not differ between the groups.

These results show that glaucoma patients with one chronic painless blind eye and a normal fellow eye with no special risk for future visual impairment experience increased depressive feelings and hopelessness for the future; experience bodily pain more frequently than the matched normal population; and underestimate their general state of health. At the same time their normal daily routine, emotional, mental and social functions do not decline. For clinical practice these results mean that these glaucoma patients perform well in their daily routine (as their ophthalmologist may experience) but their QL is reduced similar to that of the general glaucoma population. This suggests that the patients’ QL is determined by the disability of the painless blind eye, and not by the preserved visual functions of the well-functioning fellow eye.

Our study has its limitations. All our glaucoma patients and control subjects were white Europeans. Therefore, caution is needed when our results are applied to other ethnicities. The number of glaucoma patients and matched control subjects was relatively low, which is due to the relatively low number of glaucoma patients eligible to this specific study design. However, we do not think that the number of study participants represents a true limitation in the current investigation since the between-group differences were statistically and clinically significant. The design of our investigation does not allow us to differentiate between the psychological effects of losing vision on one eye, the psychological effect of being treated for glaucoma, and anxiety related to a potential functional worsening of the normal eye. This limitation, however, does not decrease the practical relevance of our results.

## Conclusion

Our results suggest that glaucoma patients with a chronic painless blind eye do suffer from increased anxiety, depression, feelings of hopelessness about their future, and do show decreased self-reported assessment of general health, even if the fellow eye’s functions are preserved with no risk of future visual impairment, and the visual functions of the fellow eye completely satisfy the patient’s visual needs. Therefore, assessing QL in glaucoma related one-eye blindness should be a task for the eye specialists. Ophthalmologists treating glaucoma patients similar to our cases need to consider providing regular psychological support to the patients. Initiation of psychological consultation may also be necessary in some cases.

## Data Availability

The datasets used and/or analyzed during the current study are available from the corresponding author on reasonable request.
